# Host–Guest Interactions of Caffeic Acid Phenethyl Ester with β-Cyclodextrins: Preparation, Characterization, and *In Vitro* Antioxidant and Antibacterial Activity

**DOI:** 10.1021/acsomega.3c07643

**Published:** 2024-01-09

**Authors:** Tayfun Acar, Pelin Pelit Arayici, Burcu Ucar, Irem Coksu, Semra Tasdurmazli, Tulin Ozbek, Serap Acar

**Affiliations:** † Bioengineering Department, Faculty of Chemical and Metallurgical Engineering, Yildiz Technical University, Istanbul 34210, Turkey; ‡ Department of Biomedical Engineering, Faculty of Engineering and Architecture, Istanbul Arel University, Istanbul 34537, Turkey; § Molecular Biology and Genetics Department, Faculty of Arts and Sciences, Yildiz Technical University, Istanbul 34220, Turkey

## Abstract

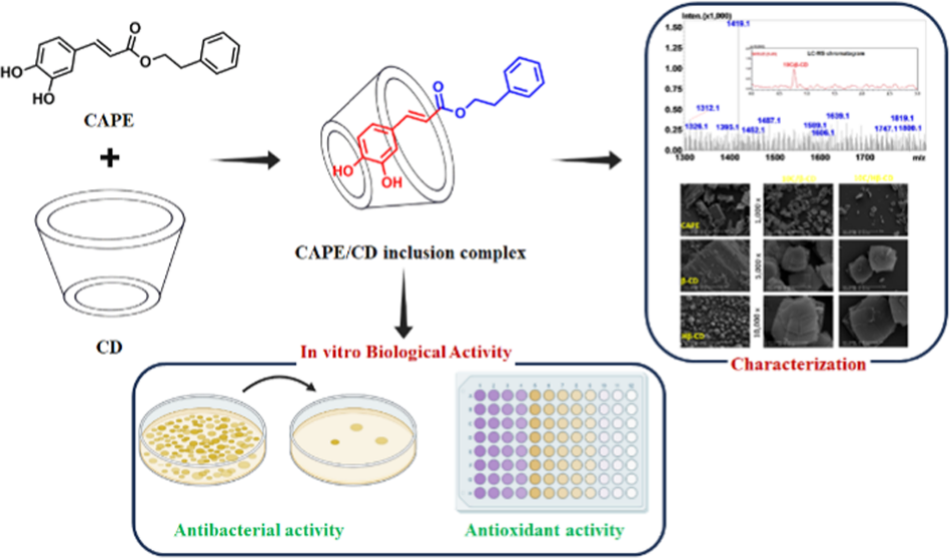

The aim of this study is to improve the solubility, chemical stability, and *in vitro* biological activity of caffeic acid phenethyl ester (CAPE) by forming inclusion complexes with β-cyclodextrin (β-CD) and hydroxypropyl-β-cyclodextrin (Hβ-CD) using the solvent evaporation method. The CAPE contents of the produced complexes were determined, and the complexes with the highest CAPE contents were selected for further characterization. Detailed characterization of inclusion complexes was performed by using Fourier transform infrared spectroscopy (FT-IR), X-ray diffraction (XRD), scanning electron microscopy (SEM), and electrospray ionization-mass spectrometry (ESI-MS). pH and thermal stability studies showed that both selected inclusion complexes exhibited better stability compared to free CAPE. Moreover, their antimicrobial activities were evaluated against *Escherichia coli* (*E. coli*) and *Staphylococcus aureus* (*S. aureus*) for the first time. According to the broth dilution assay, complexes with the highest CAPE content (10C/β-CD and 10C/Hβ-CD) exhibited considerable growth inhibition effects against both bacteria, 31.25 μg/mL and 62.5 μg/mL, respectively; contrarily, this value for free CAPE was 500 μg/mL. Furthermore, it was determined that the *in vitro* antioxidant activity of the complexes increased by about two times compared to free CAPE.

## 1 Introduction

**Figure 1:**
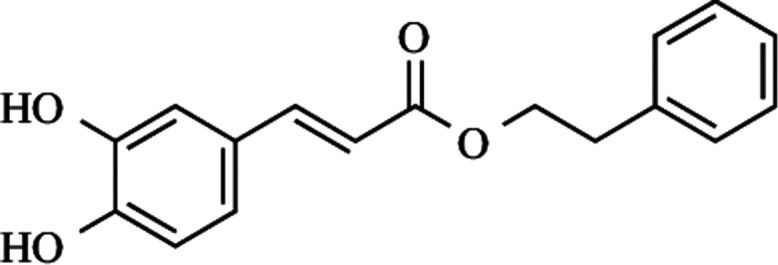
Chemical structure of CAPE.

CAPE (phenethyl 3-(3,4-dihydroxyphenyl)acrylate) is a phenolic compound with an ester bond that is easily taken into the cell due to its high cell permeability and then decomposes by intracellular esterases to release effective caffeic acid.^1^ CAPE, one of the bioactive components in propolis, is a polyphenol with hydroxyl groups in the catechol ring.^2^ Various biological properties of CAPE such as anti-inflammatory,^3^ antioxidant,^4^ antiviral,^5^ antibacterial,^6^ immunomodulatory,^7^ anticancer,^8^ and wound healing^9^ activities are due to the presence of hydroxyl groups in the catechol ring (Figure 1).^2^

In studies on the antimicrobial activity of the CAPE molecule, activity was obtained on *Enterococcus faecalis*, *Listeria monocytogenes*, *Staphylococcus aureus*, *Bacillus subtilis*, *Pseudomonas aeruginosa*, *Candida albicans*, and *Haemophilus influenzae*(6,10,11) These studies suggest that RNA, DNA, and cellular proteins are possible targets of CAPE. In addition, Takaisi-Kikuni and Schilcher suggest that the antimicrobial effect of CAPE is probably based on the inhibition of bacterial RNA polymerase.^12^ In addition, Lee et al. reported that the antimicrobial effect of CAPE is related to outer membrane damage in bacteria.^13^ In a study by Sud’ina et al., it was shown that CAPE at a concentration of 10 μM completely inhibits the formation of reactive oxygen species in human neutrophils and the xanthine/xanthine oxidase system.^14^ However, the poor water solubility (high hydrophobicity) of CAPE makes it difficult to disperse and dissolve it in aqueous systems, resulting in low bioavailability. At the same time, it has limited plasma stability and rapid clearance rate.^9,15^ To overcome these limitations, CAPE has been utilized in combination with drug delivery systems in numerous studies.^16,17^ One commonly employed approach involves the formation of inclusion complexes between hydrophobic molecules, such as CAPE, and cyclodextrins, which has been widely used to overcome these challenges.^18^

The cyclic oligosaccharides known as cyclodextrins (CDs) are generated from starch and contain 6 (α-cyclodextrin), 7 (β-cyclodextrin), 8 (γ-cyclodextrin), or more glucopyranose units linked by α-(1,4) glucosidic linkages.^19,20^ Although CDs are ring molecules, there is no free rotation at the level of bonds between the glucopyranose units. Therefore, they are not cylindrical, but toroidal or cone-shaped.^19^ The inner and outer surfaces of cyclodextrins have different polarities (a hydrophobic internal cavity and a hydrophilic external surface). Cyclodextrins are efficient transporters for hydrophobic compounds due to their inherent hydrophobic cavities. Because guest molecules penetrate the internal cavity. Due to the hydrophilic hydroxyl groups, their external surface is hydrophilic, which ensures water solubility.^21,22^ Cyclodextrins (α-, β-, and γ-CDs) are “generally recognized as safe” (GRAS) by the Food and Drug Administration (FDA).^23^

Previous studies have reported that the solubility and biological activity of CAPE are improved after combination with CDs. Garrido et al. (2018) explored the microencapsulation of caffeic acid phenethyl ester (CAPE) and caffeic acid phenethyl amide (CAPEA) through their inclusion in hydroxypropyl-β-cyclodextrin (HP-β-CD), leading to improved solubility and stability. The study demonstrates the potential of this microencapsulation technique as an effective approach for enhancing the properties of CAPE and CAPEA.^17^ The molecular properties of CAPE, a compound found in honeybee propolis with potential anticancer activity, were characterized by Wadhwa et al. (2016). It was found that the growth of cancer cells *in vitro* could be inhibited by CAPE, but its effectiveness was limited by its poor solubility in water. To address this issue, CAPE was complexed with a molecule called γ-cyclodextrin, which improved its solubility and enhanced its anticancer activity *in vitro*.^24^ Ishida et al. (2018) discussed the potential anticancer activity found in honeybee propolis, specifically focusing on the role of CAPE and its complex with γ-cyclodextrin. The study found that the complex of CAPE with γ-cyclodextrin had higher anticancer activity than CAPE alone and that this activity was due to the increased solubility and bioavailability of the complex.^25^ Although the above-mentioned studies on the inclusion phenomena of CAPE with some CD derivatives have been reported in the literature, the antioxidant and antimicrobial activities of these inclusion complexes obtained with CAPE have not been investigated. The inclusion phenomena of CAPE with β-CD were synthesized and characterized for the first time. Another novelty of this study was the performance of antioxidant and antimicrobial activity studies for the inclusion complexes of CAPE with β-CD and H-βCD.

The aim of our present study is to prepare CAPE β-cyclodextrin (β-CD) inclusion complexes using two different cyclodextrin derivatives (β-CD and hydroxypropyl-β-cyclodextrin (Hβ-CD)) through a solvent evaporation method. The objective is to enhance the solubility, stability, antioxidant, and antimicrobial activity of CAPE. The inclusion complexes were formulated with a mole ratio of 1:1 in three different reaction volumes. The CAPE content in the prepared complexes was determined, and the complex with the highest CAPE content was selected for further characterization. The inclusion complexes were thoroughly characterized by using Fourier transform infrared spectroscopy (FT-IR), X-ray diffraction (XRD), scanning electron microscopy (SEM), and electrospray ionization-mass spectrometry (ESI-MS). The stability of the complexes against pH and thermal treatments was investigated. The *in vitro* antioxidant activity of these complexes was compared with free CAPE using vitamin C as a positive control. Finally, the antibacterial activity of the complexes was tested against *E. coli* and *S. aureus*.

## 2 Materials and Methods

### 2.1 Materials

Ethanol (EtOH), β-cyclodextrin (β-CD), 2-hydroxypropyl-β-cyclodextrin (Hβ-CD), 2,2-diphenyl-1-picrylhydrazyl (DPPH), and caffeic acid phenethyl ester (CAPE) were bought from Sigma-Aldrich (St. Louis, MO). Ultrapure water was provided from the Millipore Milli-Q system.

### 2.2 Production of Inclusion Complex

Inclusion complexes were produced using the solvent evaporation method described in the literature.^26^ Six different complexes were prepared by using two different cyclodextrin derivatives, as β-CD and Hβ-CD, and three different volumes of ethanol to dissolve CAPE. For this, 5 mmol of β-CD or Hβ-CD was dissolved in 10 mL of water, and 5 mmol of CAPE was dissolved in 5 mL (5C), 10 mL (10C), and 15 mL (15C) of EtOH, respectively.

### 2.3 Characterization of Produced Inclusion Complex

#### 2.3.1 Quantification of the CAPE in the Inclusion Complexes

The amount of CAPE included in inclusion complexes was determined by a UV–vis spectrophotometer. For this, 1 mg of the complex was dissolved in 1 mL of ethanol and ultrasonicated for a while to ensure a homogeneous dispersion. The CAPE concentration in the inclusion complexes was calculated spectrophotometrically using the previously constructed CAPE standard calibration curve with absorbance measurements at 323 nm. The inclusion ratio and CAPE loading capacity were calculated as given in eqs 1 and 2, respectively.
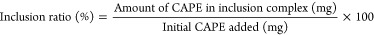
1
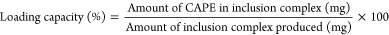
2

The reaction yield (RY) of the complexes (given as a mass percentage) was determined according to eq 3 as the ratio of the recovered mass of the produced complex to the theoretically calculated mass based on the mass of the substances used initially (CAPE+β-CD or Hβ-CD).
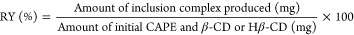
3

#### 2.3.2 Phase Solubility Study

Phase solubility studies were conducted using the technique described by Higuchi and Connors.^27^ An excess CAPE was added to aqueous solutions of β-CD in the absence and presence of CD at various concentrations (0, 2, 3, 5, 7, 9, and 10 mM). The resulting suspensions were kept at room temperature under constant stirring overnight to allow the solutions to reach equilibrium and then centrifuged for 10 min at 9000 rpm using a NÜVE NF 800R centrifuge. The supernatants were analyzed by a UV–vis spectrophotometer (UV-1700 Pharmaspec, Shimadzu, Japan). Measurements were taken in triplicate at 323 nm, and three individual measurements were averaged. The stability constant (*K*_c_) of the inclusion complex was calculated from the slope of the linear portion of the phase solubility diagram using eq 4
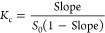
4where *S*_0_ is the solubility of CAPE in the absence of β-CD or Hβ-CD and *Slope* is the slope of the phase solubility diagram.^28^

The complexation efficiency (CE) was calculated from eq 5
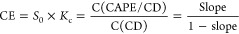
5where C(CAPE/CD) and C(CD) represent the concentrations of CAPE/β-CD or CAPE/Hβ-CD and unreacted β-CD or Hβ-CD, respectively.^29^

#### 2.3.3 Fourier Transform Infrared Spectroscopy (FT-IR) Measurements

The FT-IR measurements of CAPE, β-CD, Hβ-CD, and inclusion complexes were recorded using a PerkinElmer 1600 spectrophotometer in attenuated total reflection (ATR) mode. The FT-IR spectra, ranging from 600 to 4000 cm^–1^, were obtained with a resolution of 4 cm^–1^, and 32 scans were used.^30^

#### 2.3.4 X-ray Powder Diffraction (XRD) Measurements

The crystalline and/or amorphous structure of the CAPE, β-CD, Hβ-CD, and inclusion complexes was evaluated by X-ray powder diffraction (XRD). Powder XRD patterns of samples were analyzed at room temperature with a PANalytical X’Pert PRO powder diffractometer. The XRD spectra were recorded between 5 and 60° (2θ).

#### 2.3.5 Scanning Electron Microscopy (SEM) Analysis

The surface morphology of the inclusion complexes was evaluated with an FEI (PHILIPS) XL30 SFEG scanning electron microscope. The complexes were placed on metal surfaces and then coated with gold under vacuum and viewed with an SEM at an accelerating voltage of 15 kV.^21^

#### 2.3.6 Electrospray Ionization-Mass Spectrometry (ESI-MS) Analysis

1 mg of CD complexes was dissolved in 1 mL of water/EtOH (1:1). The mass spectra were obtained using a Shimadzu 2010 EV ESI-MS apparatus by a direct infusion method. The ESI probe voltage was set to 3 kV, the capillary temperature was maintained at 250 °C, and the nebulizer gas, N_2_ flow rate was 1.5 mL/min.^31^

#### 2.3.7 Stability Studies

The pH and thermal stability of CAPE, 10C/β-CD, and 10C/Hβ-CD were studied by taking absorbance measurements using a UV–vis spectrophotometer (UV-1700 Pharmaspec, Shimadzu, Japan).

##### 2.3.7.1 pH Stability

Pure CAPE and 10C/β-CD, and 10C/Hβ-CD inclusion complexes were dissolved with H_2_O/EtOH at a rate of 70:30. The pH was adjusted to the range of 2–12 using 0.1 N HCl and 0.1 N NaOH. The absorbance values were taken at 324 nm at 25 ± 0.1 °C.

##### 2.3.7.2 Thermal Stability

The thermal stability of free and complexed CAPE was evaluated by following the method described by Paramera et al., with some modifications.^32^ The thermal stability of CAPE, 10C/β-CD, and 10C/Hβ-CD at 60, 120, and 180 °C for 30, 60, and 120 min were examined. After thermal processing, the samples were dissolved with ethanol, and the absorbance values were taken at 324 nm at 25 ± 0.1 °C.

### 2.4 *In Vitro* Biological Studies

#### 2.4.1 Antimicrobial Activity

The antimicrobial activity of 10C/β-CD and 10C/Hβ-CD inclusion complexes was evaluated by using a broth microdilution assay, which is a quantitative method, against *E. coli* (ATCC 25922) and *S. aureus* (ATCC 25923). The antimicrobial effectiveness of 10C/β-CD and 10C/Hβ-CD was investigated in comparison with free CAPE. Additionally, whether β-CD and Hβ-CD molecules have any antibacterial effect on the bacteria was examined with the same assay. Briefly, stock solutions of CAPE, 10C/β-CD, 10C/Hβ-CD, β-CD, and Hβ-CD were prepared at a concentration of 1 mg/mL. The free CAPE sample included an equivalent amount of ingredients in the complex. The broth microdilution method was carried out according to the Clinical & Laboratory Standards Institute (CLSI) standard.^33^ The tested concentrations of the samples ranged from 500 to 31.25 μg/mL; the negative control did not contain any agent. The minimum inhibitory concentration (MIC) values of the complexes, the molecules, and the free CAPE were defined by UV–vis spectroscopy (OD_600_) and standard plate counting methods. All experiments were carried out in triplicate.

#### 2.4.2 Antioxidant Activity

Antioxidant activity was studied by DPPH radical scavenging activity protocol for CAPE, β-CD, Hβ-CD, 10C/β-CD, and 10C/Hβ-CD. Specifically, 500 μL of each sample solution in EtOH/H_2_O (1:1 (v/v)) was added to 500 μL of 0.1 mM DPPH in EtOH. The resulting solution was kept in the dark for 30 min after gentle shaking, and the absorbance was recorded at 517 nm at 25 ± 0.1 °C. The antioxidant activity assay was repeated three times for all samples and expressed as the percentage of scavenging effect and determined according to eq 6
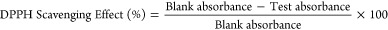
6

## 3 Results and Discussion

### 3.1 Quantification of the CAPE in the Inclusion Complexes

CAPE content and reaction yield (RY) of the six inclusion complexes obtained using the solvent evaporation method are presented in Table 1. In the production of inclusion complexes, auxiliary solvents such as EtOH, MeOH, or dichloromethane (DCM) are used for both good dissolution of the active ingredient and/or the cyclodextrin derivative.^34^ Since cyclodextrins are not dissolved in 100% EtOH, a mixture of EtOH with water is used at different rates for the production of inclusion complexes.^35^ In both cyclodextrin derivatives, the CAPE content and reaction efficiency of the complexes produced by dissolving CAPE in 10 mL of EtOH (50% (v/v) EtOH/water) was higher. More than 50% ratio use of EtOH led to a decrease of inclusion efficiency because of the reduction of CDs solubility. The effect of the ethanol/water ratio in the range of 0–100% (v/v) was researched to the complexation efficiency in a study performed by Al-Nasiri et al. in which it was aimed to form inclusion complexes of thymol, carvacrol, and linalool with β-CD. It was claimed that the EtOH ratio of the reaction environment affects importantly the complexation efficiency.^34^

**Table 1:** Inclusion Ratio, CAPE Loading Capacity, and Reaction Yield of Produced Complexes

complex code	inclusion ratio (%)	CAPE loading capacity (%)	reaction yield (%)
5C/β-CD	22.32	7.12	62.84
10C/β-CD	69.01	22.00	91.57
15C/β-CD	54.92	17.51	88.04
5C/Hβ-CD	13.30	8.90	25.29
10C/Hβ-CD	72.93	48.80	68.97
15C/Hβ-CD	61.60	41.22	68.16

In the continuation of the study, the FT-IR, XRD, SEM, and ESI-MS analyses, stability studies, and *in vitro* biological activity studies were carried out as advanced characterization studies for the above-mentioned two complexes with high CAPE content.

### 3.2 Phase Solubility Study

**Figure 2:**
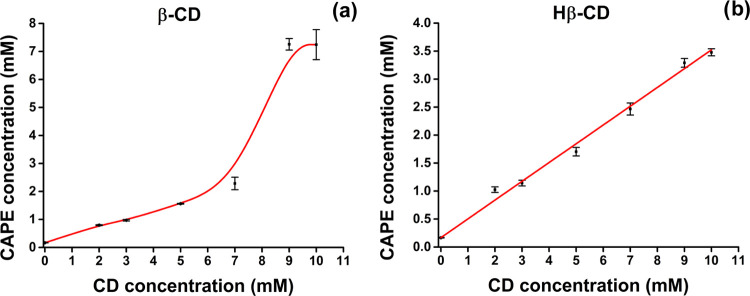
Phase solubility diagram of CAPE with different concentrations of (a) β-CD and (b) Hβ-CD. For the phase solubility study, three independent experiments were carried out. Data are shown as the mean ± standard deviation (SD) of these three separate experiments (*n* = 3).

Phase solubility studies can be used to obtain the affinity or binding constant between β-CD and CAPE. Type-A and type-B phase solubility diagrams are categorized based on how cyclodextrin and guest molecules vary in stoichiometry during inclusion complexation. The type-A phase solubility diagram includes A_L_, A_N_, and A_P_ subtypes. The A_L_ subtype model represents a linear increase in correlation between the dissolvability of guest molecules and the cyclodextrin concentration. A_N_ and A_P_ represent the positive and negative variations of the isothermal curve, respectively.^36,37^ The phase solubility diagrams of CAPE with β-CD and Hβ-CD are shown in Figure 2a,b, respectively. The aqueous solubility of CAPE increased linearly with the rising concentration of Hβ-CD over the concentration range studied. However, as the β-CD concentration increased, the solubility of CAPE in water increased faster and showed a nonlinear correlation. The phase solubility diagram of Hβ-CD can be classified as A_L_-type diagram according to the pattern proposed by Higuchi and Connors,^27^ while the phase solubility diagram of β-CD can be classified as A_P_ type. R-square (*R*^2^) value of the extrapolated curve was 0.9936 for β-CD, and that of Hβ-CD was 0.9924, indicating a strong correlation between the solubility of CAPE and cyclodextrin concentration. The calculated *K*_c_ value for β-CD was 2204.8 M^–1^, and that of Hβ-CD was 3468.2 M^–1^. It has been reported that the *K*_c_ value between 50–5000 M^–1^ is suitable for increasing the solubility and stability of hydrophobic drugs.^38^ A larger *K*_c_ value indicates a higher inclusion effect of HPβCD, which means that HPβCD has a stronger ability than βCD to increase the solubility of CAPE.^39^

CE refers to the concentration ratio between CD in an inclusion complex and free CD. In our study, the CE values were determined as 0.41 and 0.50 for the β-CD and Hβ-CD systems, respectively. The higher CE value of the Hβ-CD system (0.50) compared to the β-CD system (0.41) indicated that Hβ-CD had a higher solubilization ability for CAPE.^36^

### 3.3 FT-IR Measurements

**Figure 3:**
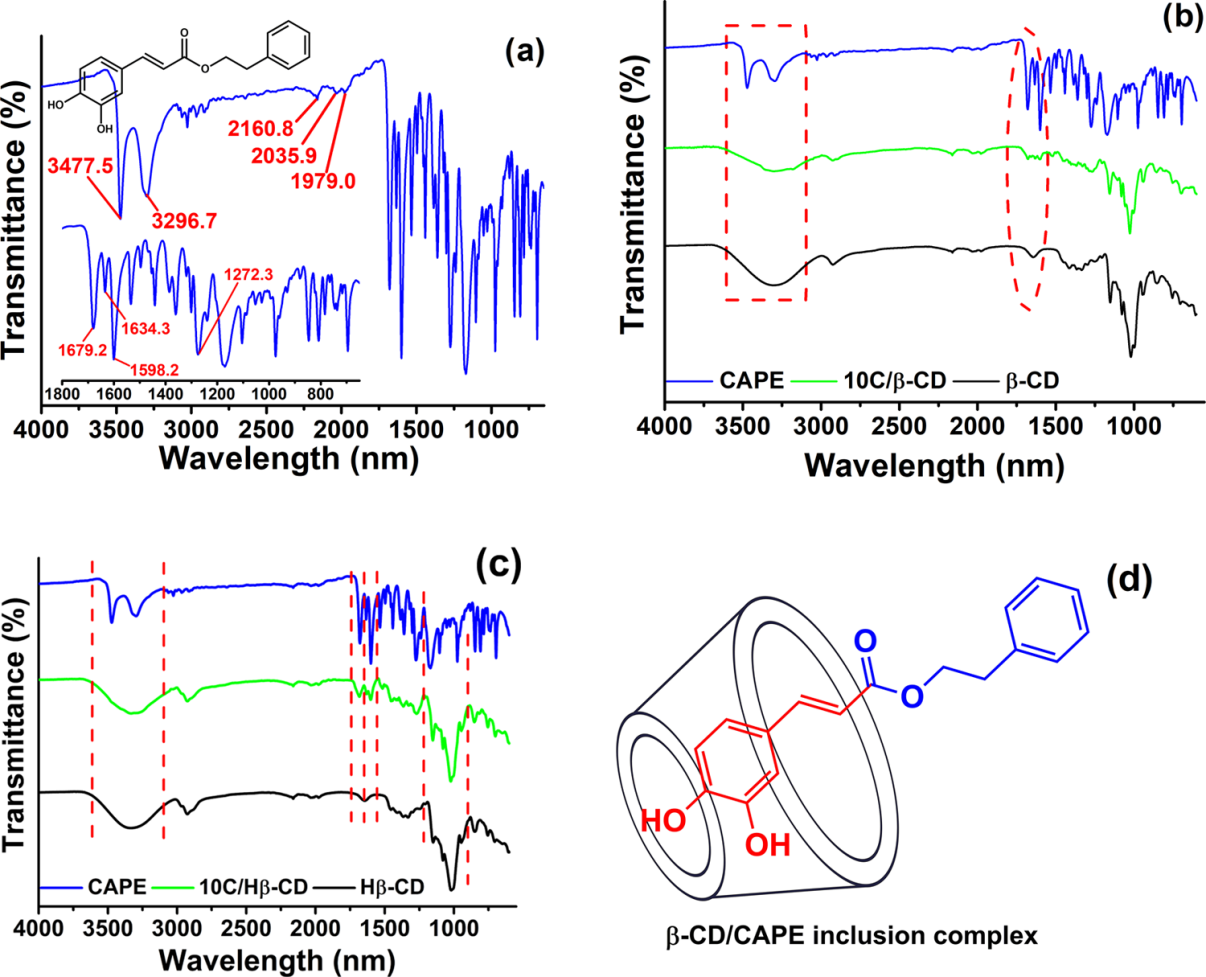
(a) FT-IR spectrum of CAPE molecule. (b) FT-IR spectrum of 10C/β-CD was given comparatively with the spectrum of CAPE and β-CD. (c) FT-IR spectrum of 10C/Hβ-CD was given comparatively with the spectrum of CAPE and Hβ-CD. (d) Recommended binding mode of CAPE with β-CD and Hβ-CD (ChemDraw Ultra 12.0 software).

The FT-IR spectra of CAPE, CD derivates, and inclusion complexes are given in Figure 3a–c. The characteristic band values of the CAPE molecule are given in Figure 3a. 3477.5 and 3296.7 cm^–1^ refer to OH groups; 2160.8, 2035.9, and 1979.0 cm^–1^ refer to aromatic C–C bonds; and 1679.2, 1598.2, and 1272.3 cm^–1^ refer to the C=O, C=C, and C–O–C groups, respectively. These bands were pretty consistent with the FT-IR spectrum of CAPE.^17^ The encapsulation of the CAPE drug by β-CD and Hβ-CD was confirmed with FT-IR spectra in Figure 3b,c, which show peaks at 1679.2 and 1598.2 cm^–1^ corresponding to C=O and C=C stretching in the drug. Moreover, the C–O–C vibration band of the CAPE at 1272.3 cm^–1^ appeared in the spectrum of both 10C/β-CD and 10C/Hβ-CD. On the other hand, the disappearance of most of the characteristic CAPE peaks in the FT-IR spectra of both inclusion complexes proved that CAPE was largely localized to the host cavity. Also, the binding mode of CAPE with CDs projected from our results is depicted in Figure 3d. This recommendation supported the proposed binding mode of CAPE with Hβ-CD in the research performed by Garrido et al. in the literature.^17^

### 3.4 XRD Measurements

**Figure 4:**
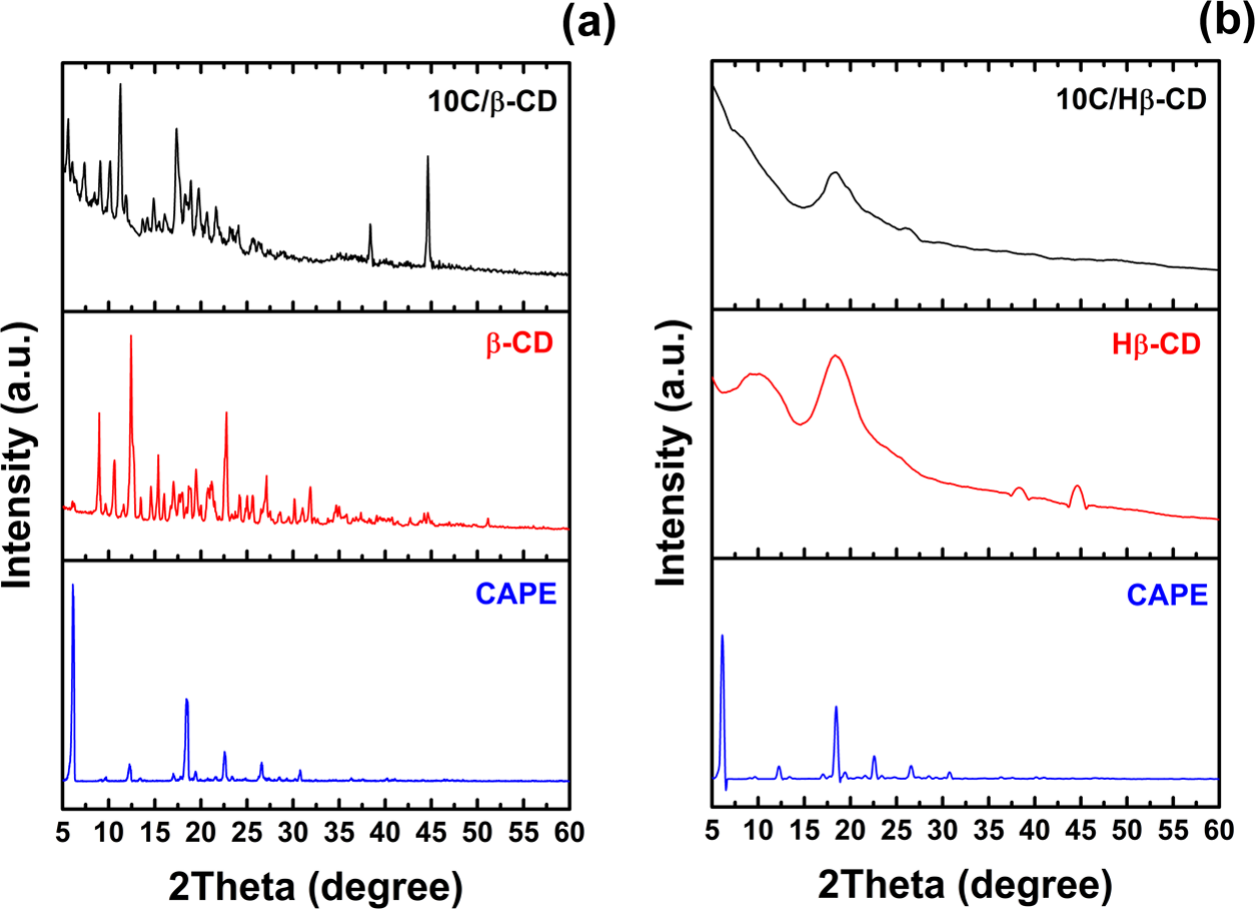
XRD patterns of (a) β-CD/CAPE and (b) Hβ-CD/CAPE comparatively given with CAPE and β-CD or Hβ-CD.

The XRD patterns of the analytes are demonstrated in Figure 4. Free CAPE showed a few sharp and narrow diffraction peaks that were characteristic of a strong crystal structure. The analysis result was consistent with the peaks defined by the literature for CAPE.^40^ β-CD displayed a series of thin and dense lines indicative of crystallinity (Figure 4a).^26^ A broad peak at 18° was sighted, appropriate with the amorphous structure of Hβ-CD (Figure 4b).^41,42^ In the 10C/β-CD inclusion complex, the characteristic peaks of CAPE have largely disappeared and the analysis result of the complex exhibited more β-CD characteristics. In addition, new peaks were formed at 37 and 44°, unlike the spectrum of free β-CD and CAPE. These changes supported the inclusion complex formation between CAPE and β-CD (Figure 4a). When CAPE was combined with Hβ-CD for the 10C/Hβ-CD inclusion complex, the crystal lattice of CAPE became disordered and its crystallinity decreased. After complexation, some characteristic diffraction peaks of CAPE disappeared (6 and 17°), and some of them were weakened. Moreover, sharp peaks of the CAPE in the range of 12–28° in the diffraction graph of the 10C/Hβ-CD inclusion complex also disappeared. A band like the broad and weak characteristic peak of Hβ-CD, supporting the complex formation and wider than the sharp peaks of CAPE, was observed at around 16.5°. In the 36–44° region, the sharp peaks of Hβ-CD disappeared, and the structure exhibited CAPE characteristics (Figure 4b). In the event, XRD analyses supported the FT-IR results discussed above for CD/CAPE complexes prepared by the solvent evaporation method. Similarly, Han et al. supported the formation of the inclusion complex between myricetin and Hβ-CD with XRD analyses.^41^ Also, the inclusion phenomena of the fluorofenidone molecule with both β-CD and Hβ-CD were studied using XRD by Wang et al.^42^

### 3.5 SEM Analysis

**Figure 5:**
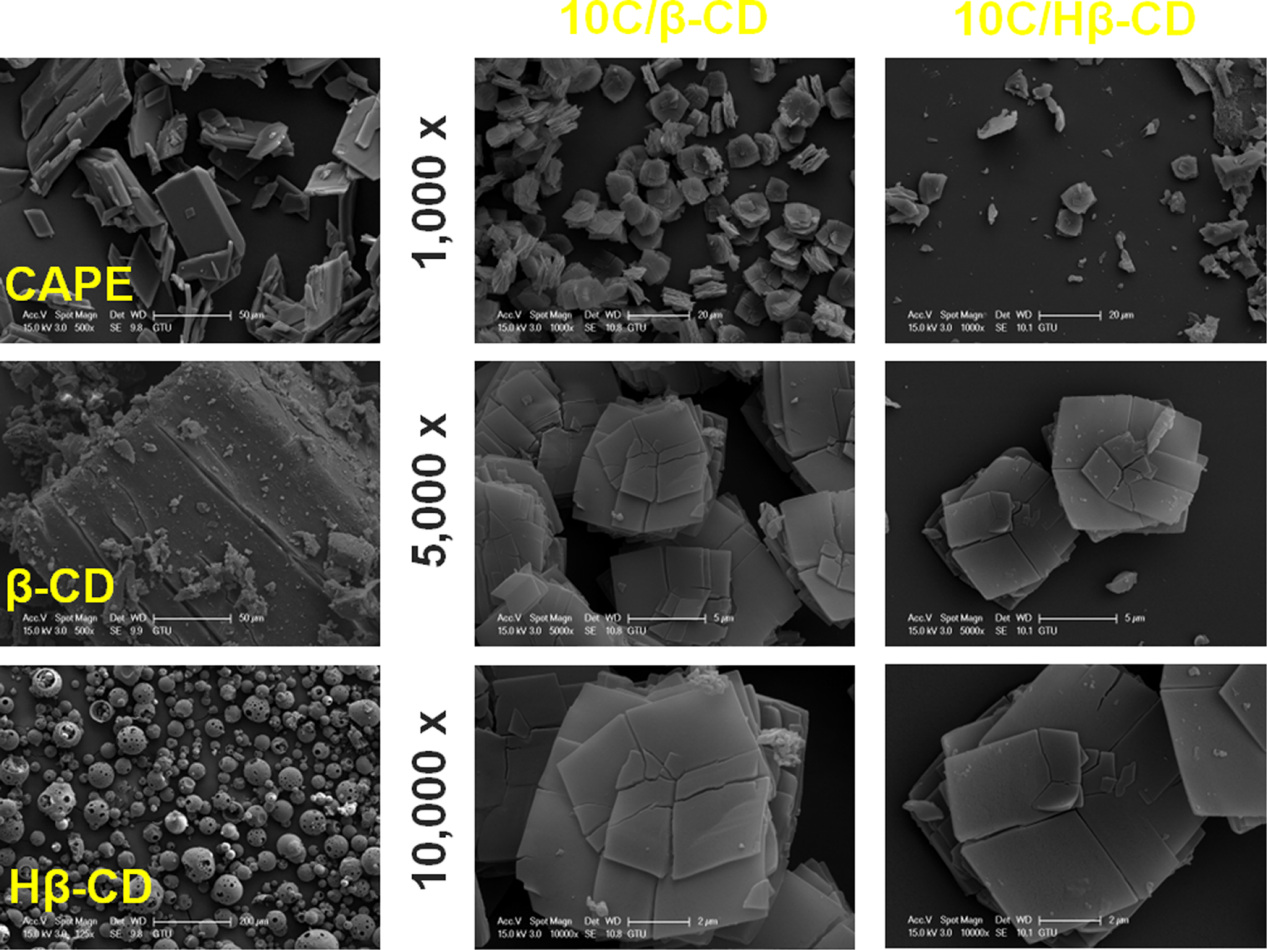
SEM micrographs of free CAPE, β -CD, Hβ-CD, and the corresponding inclusion complexes at different magnifications.

The scanning electron micrographs of the 10C/β-CD inclusion complex are shown in Figure 5. The block crystal structure of β-CD particles was like that of previous studies,^43^ while free CAPE had strip-shaped morphology. The Hβ-CD has a spherical shape with cavities on the surface.^44^ In contrast, microscopic analysis of the inclusion complexes revealed that a change had occurred in the original morphology of all three molecules (free CAPE, β-CD, and Hβ-CD). It can be seen in Figure 5 (at different magnifications) that the particles in both the 10C/β-CD and 10C/Hβ-CD inclusion complexes have a flaky structure with many lamellar crystals on the surface. This change in molecular morphology suggests the interaction between CAPE and cyclodextrins and confirms the formation of inclusion complexes. The results obtained are compatible with the literature. The nerolidol-β cyclodextrin inclusion complexes prepared by de Souza Carvalho et al.^45^ and the inclusion complex of β-acids/hydroxypropyl-β-cyclodextrin by Gu et al. showed a similar profile.^46^

### 3.6 ESI-MS Analysis

**Figure 6:**
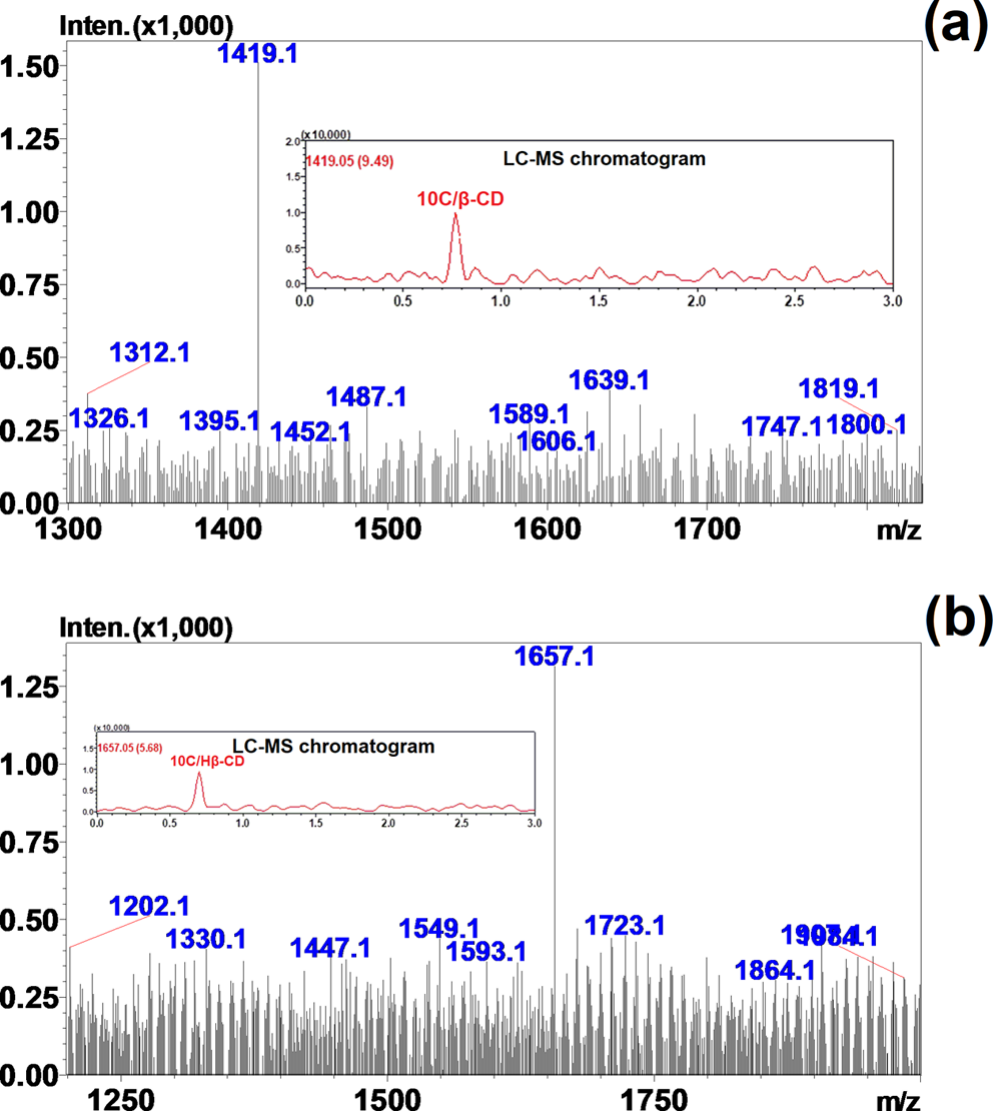
Positive-ion ESI-MS spectrum of the (a) 10C/β-CD and (b) 10C/Hβ-CD complexes verified the structure of the inclusion complex at *m*/*z* = 1419.10 and 1657.10, respectively.

Positive-ion ESI-MS was used to verify the molecular weights of the 10C/β-CD and 10C/Hβ-CD complexes. In this system, the molecular weights of CAPE, β-CD, and Hβ-CD were determined as 284.31, 1135.09, and 1374.05 Da, respectively which were completely similar to the literature.^47^ The molecular weights of the 10C/β-CD and 10C/Hβ-CD complexes were determined as [M]_obtained_ = 1419.10 Da (Figure 6a) and [M]_obtained_ = 1657.10 Da (Figure 6b), respectively. These mass values indicated that the complexation of CAPE with CDs was the 1:1 mol ratio. The 1:1 stoichiometry obtained for the 10C/Hβ-CD in the current work was suitable with the study of Garrido et al., who found a 1:1 mol ratio of CAPE/Hβ-CD in the NMR shifts.^17^

### 3.7 Stability Studies

pH and thermal stability studies of 10C/β-CD and 10C/Hβ-CD were performed comparatively with CAPE.

#### 3.7.1 pH Stability

**Figure 7:**
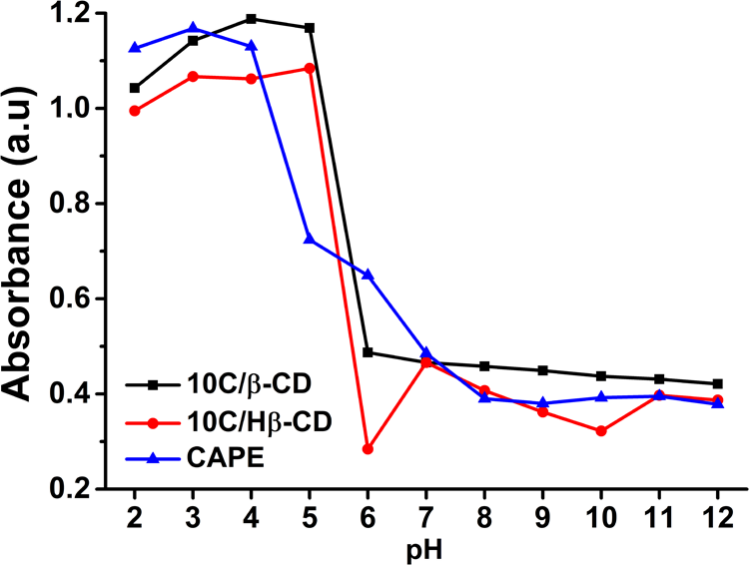
pH stability of the CAPE, 10C/β-CD, and 10C/Hβ-CD inclusion complex.

The pH stability results of CAPE, 10C/β-CD, and 10C/Hβ-CD complex are given in Figure 7. The absorbance measurements indicated that 10C/β-CD and 10C/Hβ-CD inclusion complexes show generally better pH stability compared to free CAPE. If detailed, while there was no significant decrease in the absorbance values of free CAPE, 10C/β-CD, and 10C/Hβ-CD in the extremely acidic region, the absorbance of all three types decreased rapidly as the neutral pH approached. At physiological pH and above, 10C/Hβ-CD and CAPE showed similar change graphics on absorbance values, while 10C/β-CD was slightly less affected by pH increase.

#### 3.7.2 Thermal Stability

**Figure 8:**
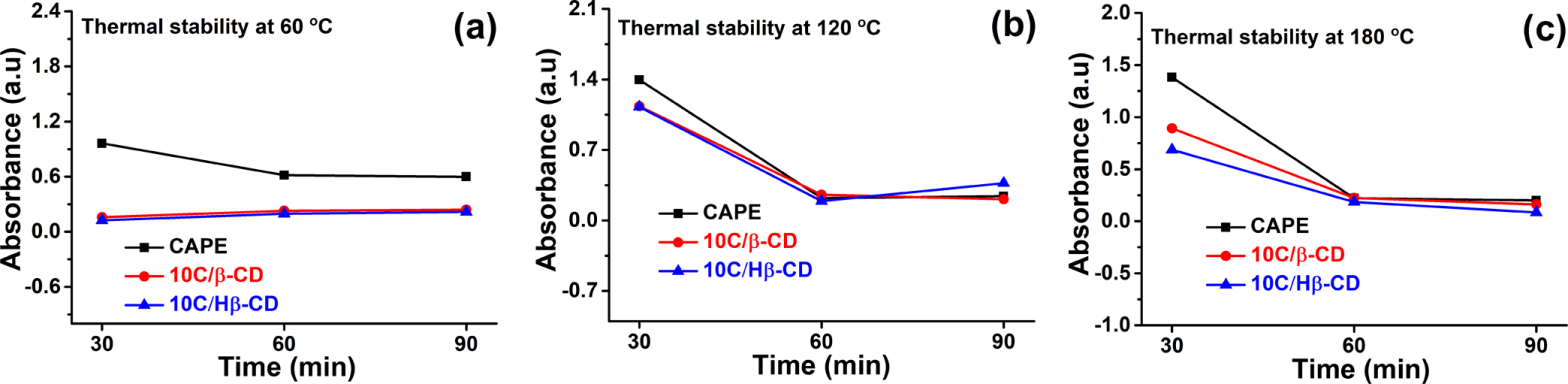
Thermal stability of the CAPE, 10C/β-CD, and 10C/Hβ-CD inclusion complexes obtained by solvent evaporation method at three different temperatures: (a) 60 °C, (b) 120 °C, and (c) 180 °C.

The thermal stabilities of pure CAPE, 10C/β-CD, and 10C/Hβ-CD inclusion complexes were assessed after 90 min isothermal heating at 60 °C (Figure 8a), 120 °C, (Figure 8b), and 180 °C (Figure 8c). Despite the decrease in the absorbances of the 10C/β-CD and 10C/Hβ-CD inclusion complexes at 60 °C compared to free CAPE, they maintained their stability at 120 and 180 °C up to 90 min. Therefore, the thermal stability of CAPE in the inclusion complex was preserved clearly when compared with intact CAPE.

### 3.8 *In Vitro* Biological Studies

#### 3.8.1 Antimicrobial Activity

**Figure 9:**
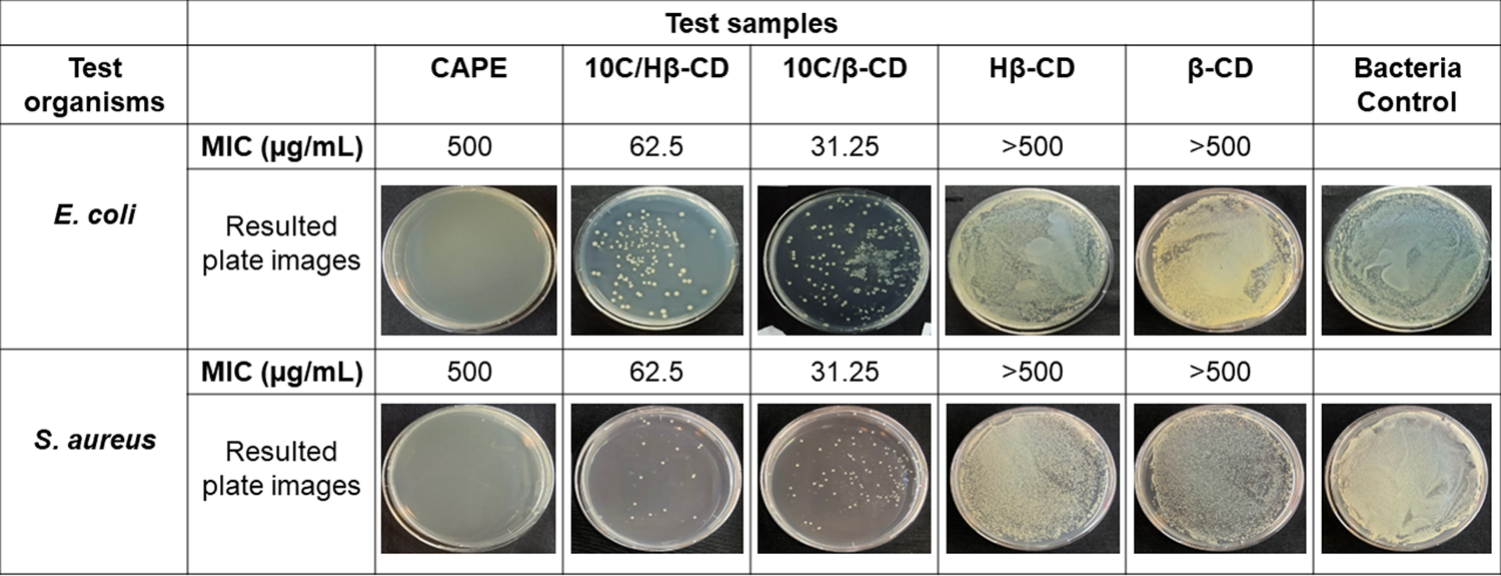
MIC values of free CAPE, Hβ-CD, β-CD, 10C/Hβ-CD, and 10C/β-CD according to Broth microdilution method and Petri dish images of the inhibition effects of samples in MIC value (diluted 4-fold).

The antimicrobial activity of the free CAPE, the inclusion complexes (10C/β-CD, and 10C/Hβ-CD), and the molecules (β-CD and Hβ-CD) was tested by the broth microdilution method where *E. coli* (ATCC 25922) and *S. aureus* (ATCC 25923) were used as Gram-negative and Gram-positive bacteria models, respectively. According to the evaluation of the assay results including spectroscopic measurement and standard plate count, MIC values were determined on both bacteria 500 μg/mL of CAPE; 62.5 μg/mL 10C/Hβ-CD; 31.25 μg/mL 10C/β-CD. It seems that the 10C/β-CD inclusion complex had better antibacterial activity than 10C/Hβ-CD. Since no effect could be seen even at the highest concentration given for β-CD and Hβ-CD, an MIC value of >500 μg/mL was expressed (Figure 9). We aimed to increase the antibacterial activity of CAPE by preparing complexes with β-CD and Hβ-CD. A quite good antibacterial effect has been achieved with the complexes designed on both bacteria, and the highest impact was obtained with 10C/Hβ-CD. It has been proven that each single β-CD and Hβ-CD molecule does not have antibacterial properties but gives a greater antibacterial character to CAPE. Although the antibacterial activity of CAPE is consistent with the literature, it may observe the changing effect level according to the methods. AlSheikh et al. (2022) found 3 mg/mL MIC on *S. aureus* using commercial CAPE and determined *S. aureus* as the most resistant among their test organisms as well as it has dose-dependent bactericidal action.^48^ In the study of Kishimoto et al. (2005),^10^ CAPE inhibited bacterial growth of *S. aureus* with 0.22–0.44 mM MIC value but not showed any antimicrobial activity on *E. coli* as well as like the other several studies^6,49^ contrary to our findings.

#### 3.8.2 Antioxidant Activity

**Figure 10:**
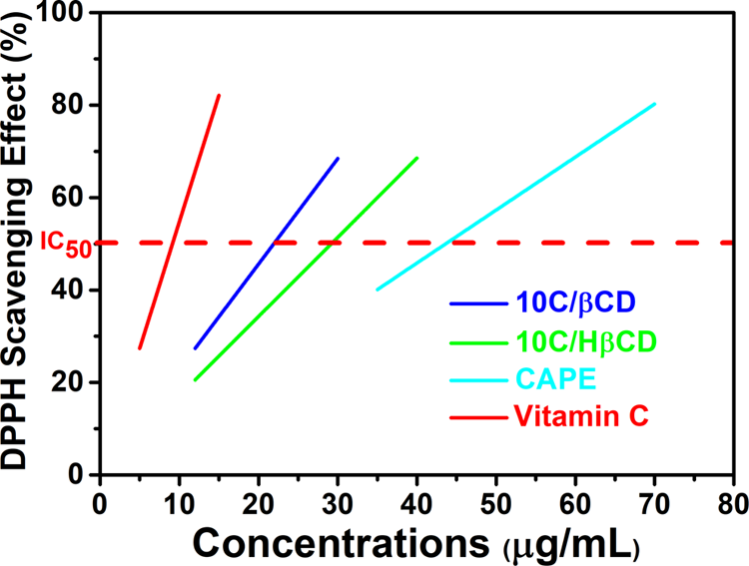
DPPH scavenging effect/final concentration of CAPE, 10C/β-CD, 10C/Hβ-CD, and vitamin C.

In general, the purpose of the *in vitro* radical scavenging essays is to provide a preliminary assessment of the antioxidant potency of the molecules and a prediction of the structure–antioxidant relationship. The DPPH radical scavenging activities of 10C/β-CD and 10C/Hβ-CD were studied by comparing them with free CAPE and the best-known antioxidant vitamin C, and the results are exhibited in Figure 10. As expected, the free cyclodextrin derivatives exhibited very low antiradical activity (data not shown) and vitamin C has the lowest IC_50_ value (9.14 μg/mL, within the accepted range in the literature for standard ascorbic acid^50^). The IC_50_ values of 10C/β-CD and 10C/Hβ-CD (21.96 and 29.18 μg/mL, respectively) were found to be lower than the IC_50_ value of free CAPE (43.65 μg/mL). The antioxidant activity of the inclusion complex is obviously differentiated from that of free CAPE. For 40 μg/mL, while the DPPH scavenging effect of CAPE is 45% it was approximately 91% and 69% for the 10C/β-CD and 10C/Hβ-CD complexes, respectively. As a result of successful complexation, the limited antioxidant activity of CAPE, as well as its water solubility, increased considerably.

## 4 Conclusions

In this study, inclusion complexes of CAPE with β-CD and Hβ-CD were produced by the solvent evaporation method in three different ratios to increase the water solubility of CAPE and to improve the antioxidant and antimicrobial efficacy of the CAPE. 10C/β-CD and 10C/Hβ-CD complexes were found as the optimum complexation ratios, and their formation was confirmed by FT-IR, XRD, and ESI-MS analyses. After that, the stability of the complexes was investigated, and more stable complexes were obtained than CAPE. While the DPPH IC_50_ values of the complexes decreased by approximately 2-fold compared to free CAPE, the complexes inhibited the growth of *S. aureus* unlike the CAPE. Hence, we determined that the synthesized inclusion complexes were effectively used to enhance the stability of CAPE as well as its antimicrobial and antioxidant activities. Consequently, the water solubility and biological activity of CAPE significantly increased, suggesting that β-CD derivatives may be beneficial in enhancing the chemical, biological, and physical properties of CAPE.
